# Titanium interlayer-mediated hydroxyapatite-coated polyetheretherketone cage in transforaminal lumbar interbody fusion surgery

**DOI:** 10.1186/s12891-021-04803-7

**Published:** 2021-11-01

**Authors:** Ce Zhu, Miaomiao He, Lili Mao, Huiliang Yang, Bowen Hu, Li Zhang, Ganjun Feng, Limin Liu, Yueming Song

**Affiliations:** 1 Department of Orthopedics Surgery and Orthopedics Research Institute, West China Hospital, Sichuan University, No. 37 Guoxue Road, Chengdu, 610041 Sichuan China; 2 Department of Spine Surgery, The Affiliated Hospital of Southwest Medical University, Luzhou, China; 3 Analytical & Testing Center, Sichuan University, Chengdu, China; 4 Department of Ultrasound, Hospital of Traditional Chinese Medicine Affiliated to Southwest Medical University, Luzhou, China

**Keywords:** Polyetheretherketone, Titanium, Hydroxyapatite, Cage, Transforaminal lumbar interbody fusion

## Abstract

**Background:**

The variance in clinical responses to polyetheretherketone (PEEK) cages with titanium (Ti) and hydroxyapatite (HA) coatings (PEEK-Ti-HA cages) is still not clear. In this study, we aimed to evaluate the radiographic and clinical outcomes of patients undergoing TLIF using PEEK-Ti-HA cages with a particular focus on fusion rate.

**Methods:**

A prospective and nonrandomized study was conducted to compare the outcomes of PEEK-Ti-HA cages (group A, *n* = 32) and uncoated PEEK cages (group B, *n* = 32). The follow up time was at least 2 years. The radiographic assessments included the regional lordosis (RL), disc height (DH), and fusion rate. The clinical indexes included the Japanese Orthopedic Association (JOA) scores and visual analog scale (VAS) scores (back and leg).

**Results:**

No significant differences were found in the pre- and postoperative RL and DH between Group A and Group B. And RL and DH, even if there were any variance initially, were restored not long after surgery in both groups. Though Group A had a significantly higher fusion rate than group B at 3 months post-surgery (93.7% vs. 75.0%), the fusion rates for the two groups reached the same level (100%) when it comes to the final follow-up. Additionally, differences of VAS and JOA scores for the two groups in general approximate.

**Conclusions:**

PEEK-Ti-HA cages, in contrast with uncoated PEEK cages, produced a better fusion rate at 3 months after single-level TLIF. The fusion rates of both groups could get 100% at the final follow-up. PEEK-Ti-HA cages could achieve similar RL, DH, JOA scores and VAS scores in comparison with uncoated PEEK cages post-surgery.

## Background

Transforaminal lumbar interbody fusion (TLIF) is a kind of surgery adopted widely in the treatment of lumbar degenerative disease (LDD). To support anterior column biomechanically and meanwhile to achieve a solid interbody fusion, various interbody cages have been designed [1]. Currently, such cages are often made of titanium (Ti) or polyetheretherketone (PEEK). Two materials that both have their advantages and disadvantages. Ti cages are found to have good biocompatibility and osteointegration ability, but their use would be limited by their radiopacity, as well as their stiffness that is higher than that of cortical bone [2, 3]. In comparison, PEEK cages would integrate advantages like satisfying physical and chemical stability, stiffness approximating native bone, and excellent radiolucency [2]. PEEK cages’ properties as these would greatly help, in postoperative imaging evaluations, to prevent stress shielding and interference, which are often observed in the cases adopting Ti cages. With this merit, however, the osseointegration capacity of PEEK cages is far from satisfaction due to their bioinert surface.

So far, two remedial measures would be potentially taken to enhance the osseointegration capacity of PEEK cages: one is the synthesis of PEEK and composites with bioactive materials, and the other is the modification of the PEEK surface [4]. However, while the addition of other possible composites could indeed enhance the osseointegration capacity of PEEK cages, the PEEK’s original mechanical properties might be reduced largely. By contrast, surface modifications can not only improve the bone-binding ability but also preserve the mechanical properties of PEEK [5, 6].

Hydroxyapatite (HA), an inorganic bioceramic constituent of human bones and teeth, possesses high levels of biocompatibility, osteoinductivity, and bioactivity, and a large surface area [7]. When employed to coat the surfaces of PEEK components, it has demonstrated exciting efficacy in improving the PEEK’s bone-binding ability [8, 9]. In addition, several coating techniques pertinent to the application of HA, including plasma spraying, sputtering, arc ion plating and microwave-assisted deposition, have also been reported [4]. Among those techniques, plasma spraying, with its merits of high repeatability and deposition rates, has been widely adopted for commercial use [4, 10]. However, the adhesion strengths of plasma-sprayed HA coatings were found to be very low and had the tendency of delaminating [9]. To enhance the level of adherence, a new cage was prepared in this study: Ti layer was firstly created on the PEEK surface via plasma spraying, and then on the Ti player another layer of HA film was sprayed. This new cage was expected to improve the quality and rate of interbody fusion in spinal interbody fusion surgery to ensure positive long-term clinical outcomes.

In one of our previous studies, we adopted PEEK-Ti-HA cages in anterior cervical discectomy and fusion (ACDF), finding that in comparison with uncoated PEEK cages, PEEK-Ti-HA cages demonstrated a higher fusion rate at 3 months post-surgery [11]. However, since there had not been many studies yet focusing on clinical responses to PEEK-Ti-HA cages, we further introduced PEEK-Ti-HA cages into TLIF for the treatment of single-level LDD. In this study, we aimed to evaluate the radiographic and clinical outcomes of patients undergoing TLIF using PEEK-Ti-HA cages with a particular focus on fusion rate.

## Methods

This study was approved by the ethics committee of West China Hospital of Sichuan University and informed consent was obtained from the patients. All methods were carried out in accordance with relevant guidelines and regulations.

**Fig. 1:**
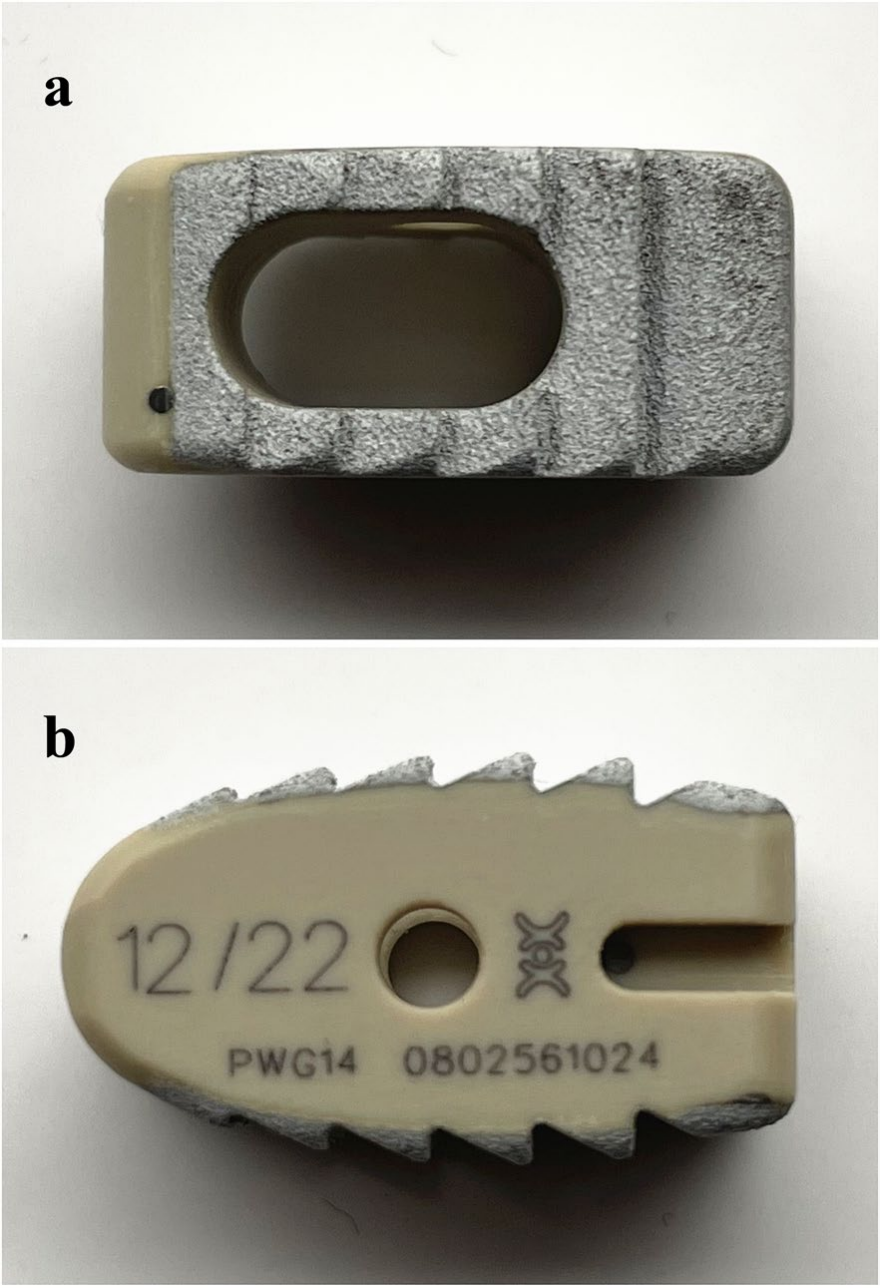
Horizontal view (**a**) and lateral view (**b**) of the PEEK-Ti-HA cage

PEEK-Ti-HA cages employed in this study were manufactured via plasma spraying technique by WEGO Holding Co., Ltd. China (See Fig. 1 for the horizontal and lateral views of the PEEK-Ti-HA cage). Scanning electron microscopy (SEM, JSM-7500F, JOEL, Japan) was used to photograph the top and cross-sectional views of the cage. Line energy-dispersive spectrometry (EDS, JSM-7500F, JOEL, Japan) was used to analyze the elements of PEEK, Ti, and HA. The surface phases of the cage were further recorded by X-ray diffraction (XRD, EMPYREAN, PANalytical B.V., Holland) and Fourier transform infrared spectroscopy (FTIR, Nicolet 6700, US). Compressive test was carried out by using a universal mechanical testing machine (MTS, model E45, America) and the loading speed was set at 4 mm/min.

A prospective and nonrandomized study was conducted to compare the outcomes of TLIF employing PEEK-Ti-HA cages (group A) and uncoated PEEK cages (group B). Patients with single-level LDD enrolled in this study were those who received TLIF between August 2016 and October 2017. All these patients had been given non-surgical managements such as pharmacotherapy and physiotherapy for at least 3 months before receiving TLIF, but no efficacy was seen. The exclusion criteria were as follows: suppurative spondylitis, ankylosing spondylitis, spinal tuberculosis, spinal tumors, and previous spine surgery. The follow-up time was at least 2 years.

TLIF surgery was performed using the conventional open technique by a single surgeon under general anesthesia [12]. The suitable cage filled with local bones was implanted to the intervertebral space after decompression. Then, the pedicle screws and rods (CD HORIZON LEGACY System, Medtronic, USA) were inserted to the vertebra for stabilization. Patients could get out of the bed at day 2 after the surgery. After surgery, the patients wore braces while walking for approximately 3 months.

**Fig. 2:**
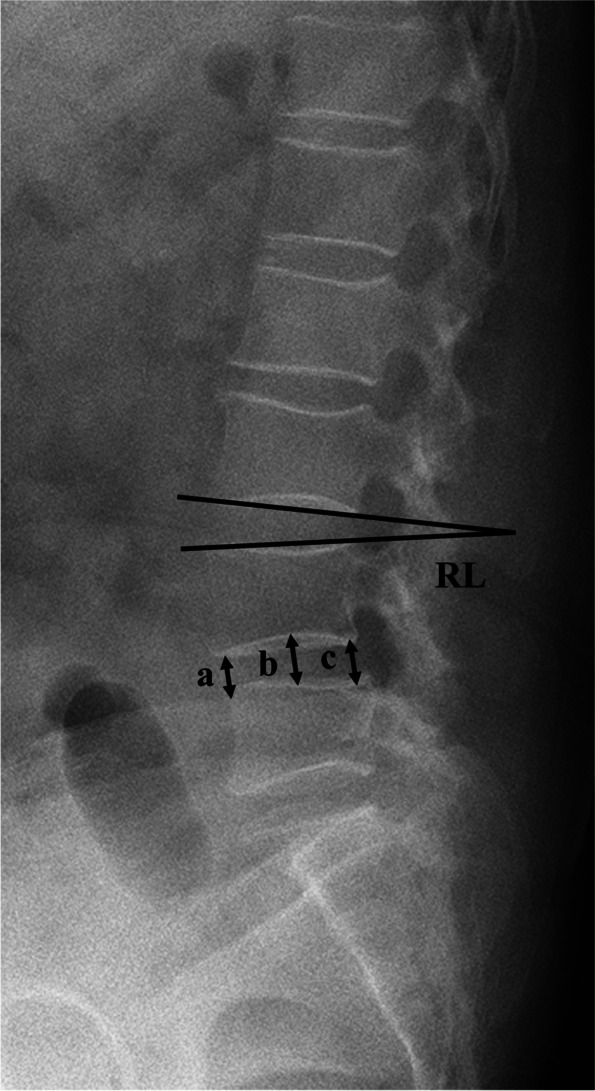
Radiographic measurements: RL (regional lordosis), the angle between the upper and lower edges of the intervertebral disc; DH (disc height), the mean of the anterior (**a**), middle (**b**), and posterior disc height values (**c**)

Plain radiographs and CT scanning of the lumbar spine were taken preoperatively, 3 months post-surgery and at the final follow-up. The radiographic assessments included (Fig. 2): regional lordosis (RL), the angle between the upper and lower edges of the intervertebral disc; disc height (DH), the average value of the anterior, middle, and posterior disc heights. Kyphosis was calculated as negative, while lordosis was calculated as positive. The loss of DH more than 3 mm was defined as subsidence [13]. Whether fusion was achieved was determined on CT scanning [14]: **unfused, grade 1** (*Obvious radiographic pseudarthrosis based on collapse of the construct, loss of disc height, vertebral slip, broken screws, displacement of the cage.*) **or grade 2** (*Probable radiographic pseudarthrosis based on significant resorption of the bone graft or a major lucency or gap visible in the fusion area.*); **uncertain, grade 3** (*Bone graft is visible in the fusion area at approximately the density originally achieved surgically. A small lucency or gap may be visible involving just a portion of the fusion area with at least half of the graft area showing no lucency between the graft bone and vertebral bone.*); **fused, grade 4** (*Bone bridges the entire fusion area with at least the density originally achieved at surgery. There should be no lucency between the donor bone and vertebral bone.*) **or grade 5** (*The bone in the fusion area is radiographically denser and more mature than originally achieved in surgery. And no lucency could be detected between the graft bone and cage with vertebral bone.*). Additional lateral flexion and extension lumbar radiographs were obtained at the final follow-up to assess the range of angular motion. Fusion was defined when an angular motion of < 5° and translational motion of < 3 mm were present [15, 16].

At 3 months post-surgery and the final follow-up, both Japanese Orthopedic Association (JOA) scores and visual analog scale (VAS) scores (back and leg) were introduced to assess clinical outcomes.

SPSS software (version 22.0; IBM Corp., USA) was used for statistical analysis. The continuous data were expressed as means ± standard deviations (SDs) and analyzed by using Student’s t-test. The categorical data were compared by using the chi-square test or Fisher’s exact test appropriately. *P <* 0.05 was considered significant.

## Results

**Fig. 3:**
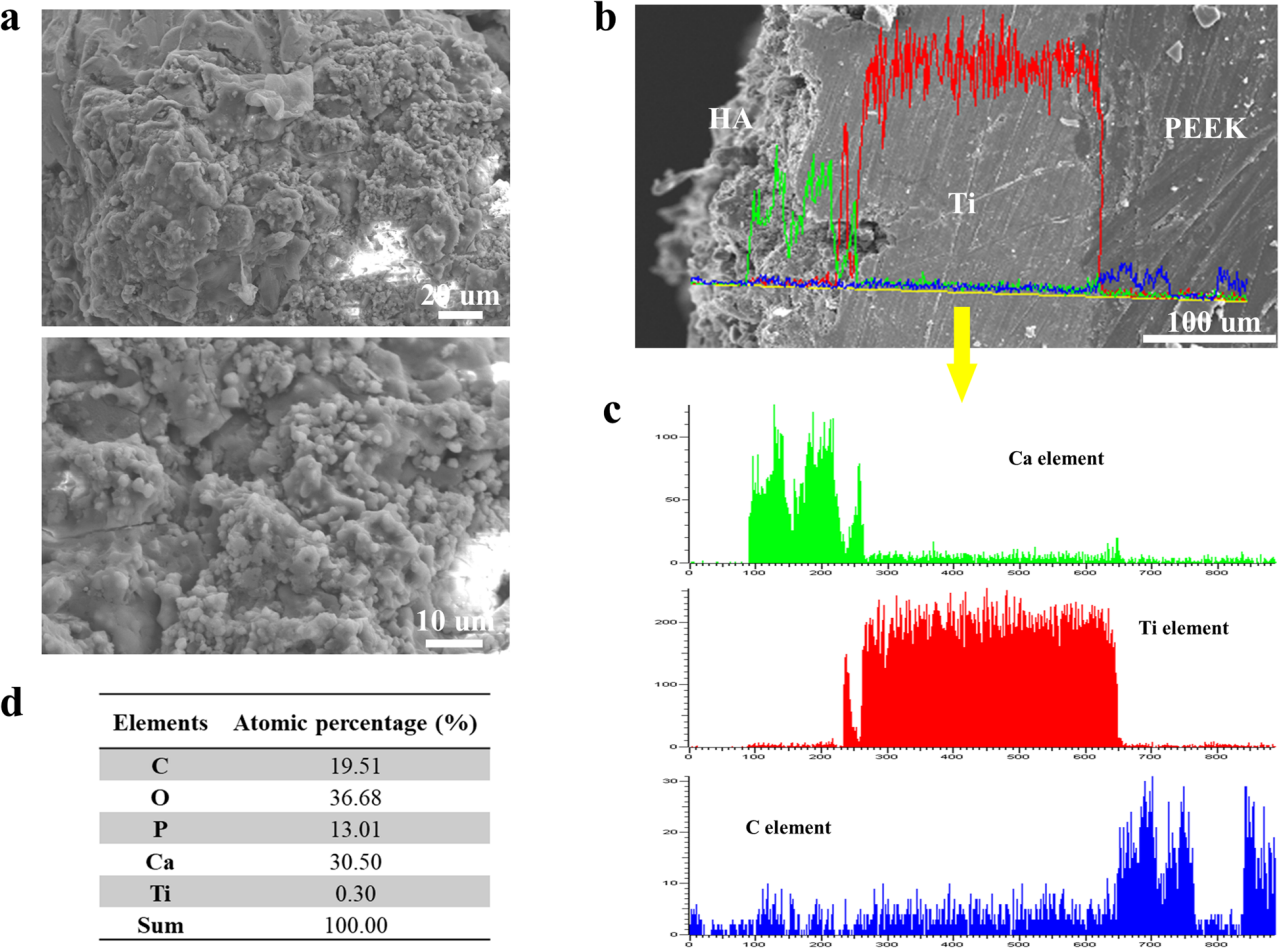
**a** Surface SEM images of the PEEK-Ti-HA cage; **b** cross-sectional SEM images; **c** corresponding EDS line scanning; **d** element percentage of PEEK-Ti-HA

The morphology of the surface of PEEK-Ti-HA cages was shown in Fig. 3a. HA particles with a size of 2 μm were sprayed on the surface of the PEEK cage. The cross-sectional view of the PEEK cage, as well as its corresponding EDS line, was displayed in Fig. 3b, and it can be seen from Fig. 3c that the original surface of the PEEK cage was coated first with Ti and then on top of it another layer of HA. The C element originated from the PEEK substrate, the intermediate area was Ti, and the Ca element originated from HA, indicating that HA was mainly focused on the surface of the PEEK cage. Specifically, the overlapping area between the C and Ti elements indicated that the plasma spraying technique realized a certain depth of metallic penetration on the polymer substrate for strong bonding at the interface. Figure 3d shows the percentage of different elements on the surface of the PEEK cage.

**Fig. 4:**
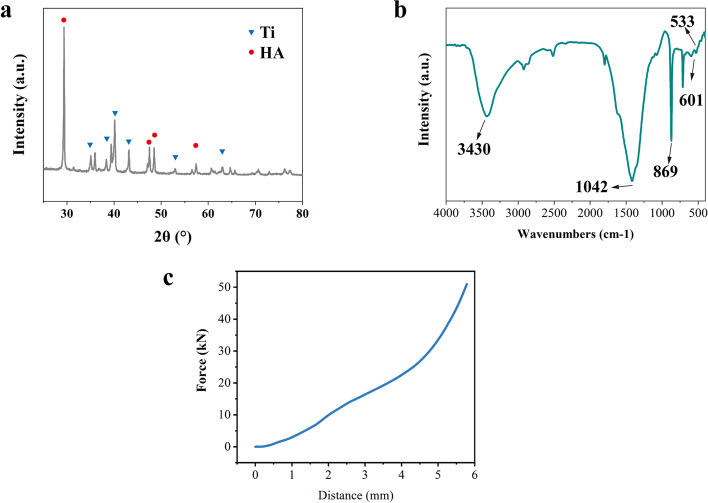
XRD (**a**) and FTIR (**b**) of PEEK-Ti-HA; (**c**) the force-distance curve of PEEK-Ti-HA

The investigation of XRD spectrum of the PEEK was listed in Fig. 4a. The diffraction peaks at 28.9°, 48.6°, and 48.01° were the crystal plane of 210, 320 and 312 respectively, indicating successful preparation of HA (PDF# 09-0432) by plasma spraying technique. The characteristic peak of Ti was also confirmed which is corresponded with the card of PDF# 44-1294. The XRD analysis indicated the main compositions of the coating were HA and Ti. FTIR of the cage surface was performed, and the findings are shown in Fig. 4b. The characteristic peak at 3430 cm ^− 1^ was -OH. The stretching vibration peak at 1042 cm^− 1^ was attributed to -PO4, while 601 cm^− 1^ was its bending vibration peak, which indicated the existence of HA. However, Ti did not have an obvious characteristic FTIR peak owing to the nonpolar bond. The results of compressive test (Fig. 4c) indicated that the cage deformed with forces higher than 10kN, which meets the mechanical requirements of the spine.

Sixty-four patients in total (Group A: Group B = 32: 32) were enrolled in this study. The mean follow-up duration was 29.7 ± 7.1 months (24 ~ 47 months). The differences in age, sex, smoking status, operative segments, operation time, or intraoperative blood loss between Group A and Group B were not significant (Table 1).

**Table 1:** Demographic data

	Group A (***n*** = 32)	Group B (***n*** = 32)	***P***
**Age (years)**	54.5 ± 6.9	55.3 ± 7.2	0.660
**Male: Female**	20: 12	17: 15	0.613
**Smoker: Non-smoker**	11: 32	14: 32	0.609
**Operative Levels**			
L3/4	8	6	
L4/5	16	17	0.829
L5/S1	8	9	
**Operation Time (min)**	193.0 ± 25.7	201.2 ± 24.2	0.193
**Intraoperative Blood Loss (mL)**	372.8 ± 107.0	378.7 ± 115.2	0.832

*P < 0.05

The radiological outcomes of the patients in both groups were listed in Table 2. There were no significant differences between Group A and Group B regarding RL and DH before and after the surgery. Table 3 exhibited the clinical outcomes of TLIF in the Group A and Group B. No significant differences were found regarding the JOA or VAS scores before and after surgery between the two groups (*P* > 0.05).

**Table 2:** Pre- and postoperative radiological parameters

	Group A (***n*** = 32)	Group B (***n*** = 32)	***P***
**Regional Lordosis (**°**)**			
Pre-operation	6.1 ± 2.9	5.6 ± 2.9	0.531
3 months	12.6 ± 1.5*	12.3 ± 1.0*	0.363
Final Follow-up	12.7 ± 1.4*	12.1 ± 1.1*	0.077
**Disc Height (mm)**			
Pre-operation	6.4 ± 1.5	6.0 ± 0.9	0.282
3 months	12.4 ± 1.8*	11.7 ± 1.2*	0.080
Final Follow-up	11.5 ± 1.6*#	11.0 ± 1.1*#	0.223
**Fusion Rate**			
3 months	93.7% (30/32)	75.0% (24/32)	0.039
Final Follow-up	100%	100%	/

*P < 0.05 compared with pre-operation

#*P* < 0.05 compared with 3 months

**Table 3:** Pre- and postoperative clinical indexes

	Group A (***n*** = 32)	Group B (***n*** = 32)	***P***
**JOA score**			
Pre-operation	14.9 ± 2.9	15.2 ± 2.5	0.649
3 months	25.5 ± 1.5*	25.6 ± 1.4*	0.676
Final Follow-up	25.8 ± 1.2*	26.0 ± 1.1*	0.466
**VAS back**			
Pre-operation	6.6 ± 1.5	6.8 ± 1.7	0.651
3 months	2.6 ± 1.0*	3.0 ± 1.2*	0.215
Final Follow-up	2.3 ± 0.9*#	2.3 ± 0.9*#	0.798
**VAS leg**			
Pre-operation	6.6 ± 1.4	6.7 ± 1.2	0.781
3 months	2.3 ± 0.9*	1.9 ± 1.1*	0.194
Final Follow-up	2.0 ± 0.9*	1.7 ± 1.0*	0.253

*P < 0.05 compared with pre-operation

#*P* < 0.05 compared with 3 months

## Discussion

Many experimental and clinical studies have proved that PEEK with Ti and/or HA coatings can achieve better biological properties and preserve the suitable biomechanical properties of PEEK at the same time [4, 10]. Liu et al. [17] reported that the Ti-PEEK cages could improve cell proliferation and expression of osteogenic gene/protein of mouse MC3T3-E1 pre-osteoblasts in vitro, and also enhance bone formation activity of PEEK in vivo sheep tests. The introduction of HA coating on PEEK could activate higher ALP activity and extracellular matrix mineralization of human bone marrow mesenchymal stem cells, and also enhance the early bone response to PEEK implants in vivo animal models [18, 19]. Clinically, Ti-coated PEEK cage can help the bone around the cage to grow better than uncoated PEEK cage and finally promote solid fusion and improvement of clinical outcomes of both PLIF and TLIF [2, 20, 21]. However, the clinical studies of HA-coated spinal cages were still lacking. Likewise, to our knowledge, the published data of Ti-HA PEEK cages were also limited. In the present study, we aimed to evaluated the efficacy of PEEK-Ti-HA cages in comparison with uncoated PEEK cages in patients with single-level TLIF.

**Fig. 5:**
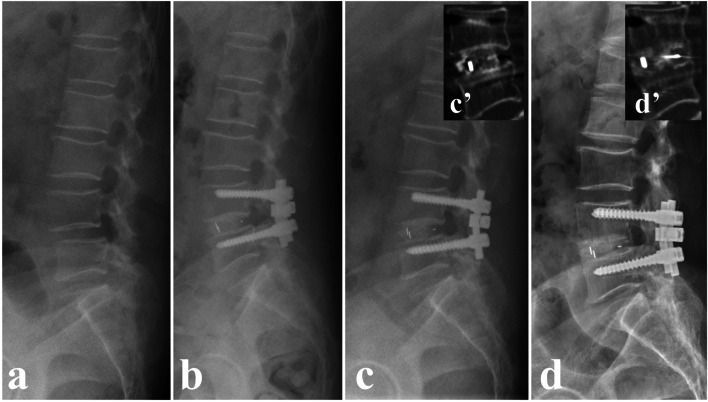
A 53-year-old woman underwent TLIF with a PEEK-Ti-HA cage. **a** Preoperative X-ray. **b** Postoperative X-ray. **c** X-ray at 3-month post-surgery. **c’** CT scanning at 3-month post-surgery. **d** X-ray at the final follow-up. **d’** CT scanning at the final follow-up

Successful lumbar fusion results can lead to early postoperative rehabilitation and predict satisfactory clinical outcomes [2, 22]. In this study, Group A had a significantly higher fusion rate than group B at 3 months post-surgery (93.7% vs. 75.0%, *P* = 0.039), while the fusion rates for the two groups could achieve 100% at the final follow-up. The results are mainly due to the favorable osseointegration ability of Ti and HA coatings on the PEEK cages: HA is an osteoconductive bioceramic material which has similar mineral phase with native bone. It possesses excellent biocompatibility and promotes the integration of implants and the surrounding bone [19]. Ti coatings can also facilitate the growth of bone tissues due to its good biocompatibility and osteoconductivity [23, 24]. Moreover, the plasma-sprayed Ti coating can provide a 6-12 μm roughness surface with multiple pores, which provides original fixation of the intervertebral area by limiting micromotion and enhancing the frictional forces [3, 20]. The combination of Ti and HA in the coatings can achieve favorable biological results in vivo (Fig. 5), which are in accordance with the outcomes of a sheep pelvic model reported by Stübinger et al. [25].

Subsidence is a usual postoperative complication following TLIF and is associated with fusion failures [2]. In the present study, the cage subsidence rate was comparable between Group A and Group B (3/32, 9.3% vs. 2/32, 6.2%; *P* = 0.641). The DH in the Group A restored from 6.4 mm to 12.4 mm after surgery but reduced to 11.5 mm at the final follow-up, while the DH in Group B restored from 6.0 mm to 11.7 mm after surgery but reduced to 11.0 mm at the final follow-up (Table 2). The mean magnitudes of loss in DH of the operative segment were similar between the two groups (Group A: Group B = 0.9 mm: 0.7 mm, *P* = 0.577). There were five patients who had cage subsidence at the final follow up. But fortunately, all of them had no relevant clinical symptoms and achieved solid osseous fusion at the final follow up.

As an important component of global sagittal alignment, the loss of lumbar lordosis after lumbar spine fusion surgery is strongly correlated with chronic low back pain (CLBP), degenerated adjacent segments, and poor health-related quality of life (HRQOL) [26–28]. In this study, RL of patients in both groups restored significantly after surgery and remained stable until the final follow-up (Table 2). Furthermore, the differences in RL between the Group A and Group B preoperatively and at the 3-month and final follow-up visits were not statistically significant.

The clinical outcomes of Group A and Group B were comparable before and after the surgery. In both groups, the JOA scores and VAS leg scores were seen rising up drastically after surgery, and such effect maintained at the final follow-up. In addition, the VAS back scores of both groups improved 3 months after surgery and increased to even greater ones at the final follow-up.

However, the results of our study need to be interpreted with consideration of its limitations. Since this study only examines statistics from a single center, its sample size was too small. Additionally, while its early data are encouraging, the follow-up duration, if possible, should have been much longer, so as to obtain more convincing statistics tested by time. In the meantime, we did not include the discussion of the clinical outcomes of patients with multilevel LDD. Thus, in the future research, it might be ideal that patients with multilevel LDD would also be enrolled and longer follow-up periods would be adopted. Furthermore, PEEK-Ti-HA cages’ cost-effectiveness and their application of PEEK-Ti-HA cages would also be explored in patients who are at a higher risk of pseudoarthrosis (senior patients with osteoporosis, for instance).

## Conclusions

In contrast with those uncoated PEEK cages, PEEK-Ti-HA cages produced a markedly higher fusion rate at 3 months after single-level TLIF. The fusion rates of both groups were able to arrive at 100% at the final follow-up. PEEK-Ti-HA cages could also achieve RL, DH, JOA scores and VAS scores similar to that the uncoated PEEK cages yield post-surgery.

## Data Availability

Data will be available upon request to the corresponding author.

## Abbreviations

PEEK: Polyetheretherketone
Ti: Titanium
HA: Hydroxyapatite
TLIF: Transforaminal lumbar interbody fusion
LDD: Lumbar degenerative disease
ACDF: Anterior cervical discectomy and fusion
DH: Disc height
RL: Regional lordosis
VAS: Visual analog scale
JOA: Japanese Orthopedic Association
SEM: Scanning electron microscopy
EDS: Energy-dispersive spectrometry
XRD: X-ray diffraction
FTIR: Fourier transform infrared spectroscopy
MTS: Mechanical testing machine
CT: Computerized tomography
HRQOL: Health-related quality of life
